# Predicting the Potential Spread of *Diabrotica virgifera virgifera* in Europe Using Climate-Based Spatial Risk Modeling

**DOI:** 10.3390/insects16101005

**Published:** 2025-09-27

**Authors:** Ioana Grozea, Diana Maria Purice, Snejana Damianov, Levente Molnar, Adrian Grozea, Ana Maria Virteiu

**Affiliations:** Faculty of Agriculture, University of Life Sciences “King Mihai I” from Timișoara, Calea Aradului 119, 300645 Timisoara, Romania

**Keywords:** western corn rootworm, invasive pest species, maize agroecosystems, thermal threshold, Europe, pest surveillance, spatial modeling, pest range expansion

## Abstract

*Diabrotica virgifera virgifera* Le Conte, 1868 (Coleoptera: Chrysomelidae), known as the western corn rootworm, is one of the most important alien insect pests affecting maize crops globally. It causes significant economic losses by feeding on the roots, which affects plant stability and nutrient absorption, as well as by attacking essential aerial organs (leaves, silk, pollen). Since its accidental introduction into Europe, the species has expanded its range across maize-growing regions, raising concerns about future distribution under climate change. This study aimed to estimate the risk of pest establishment across Europe over three future time frames (2034, 2054, 2074) based on geographic coordinates, climate data, and maize distribution. Spatial simulations were performed in QGIS using national centroid datasets, risk classification criteria, and temperature anomaly maps derived from Copernicus and ECA&D databases for 1992–2024. The results indicate consistently high risk in southern and southeastern regions, with projected expansion toward central and western areas by 2074. Risk zones showed clear spatial aggregation and directional spread correlated with warming trends and maize availability. The pest’s high reproductive potential, thermal tolerance, and capacity for human-assisted dispersal further support these predictions. The model emphasizes the need for expanded surveillance in at-risk zones and targeted policies in areas where *D. v. virgifera* has not yet established. Future work should refine spatial predictions using field validation, genetic monitoring, and dispersal modeling. The results contribute to anticipatory pest management planning and can support sustainable maize production across changing agroclimatic zones in Europe.

## Simple Summary

*Diabrotica virgifera virgifera* Le Conte is a highly destructive beetle from the Chrysomelidae family that attacks maize roots, weakening plants and causing serious yield losses. Originally from North America, it has spread across Europe, especially in warm regions where maize is widely cultivated. In this study, we assessed the pest’s potential to expand into new areas based on geography, maize availability, and climate conditions. A spatial model estimated risk levels for different parts of Europe and showed a clear trend of expansion toward cooler regions. The pest’s ability to reproduce rapidly and tolerate environmental changes increases the threat to future crops. These results can help guide surveillance programs and inform decisions on pest management to protect maize production and ensure food security.

## 1. Introduction

*Diabrotica virgifera virgifera* (Le Conte, 1868), known as Western corn rootworm, a member of the leaf beetle family (Chrysomelidae) within the order Coleoptera, first appeared in Europe approximately 33 years ago, near Belgrade, Serbia [1]. Since its introduction, the species has gradually expanded its range and is now established across several parts of the continent. Its presence has been confirmed in Eastern and Southeastern Europe: Serbia, Romania, Hungary, Bulgaria, Ukraine, Belarus, Rusia, Greece, Bosnia and Herzegovina [2,3,4,5,6,7,8,9,10,11,12], Central Europe: Austria, Czech Republic, Slovakia, Poland [13,14], Western Europe: Italy, France, Germany, Switzerland, Spain [15,16,17,18,19,20] and other regions: parts of the Benelux and Baltic countries [21]. In any case, the expansion clearly centers around the initial point of detection.

The history of this species in Europe is quite remarkable. Since its first detection in Europe, the insect, long regarded as an invasive threat [22], has followed a dynamic course involving geographic expansion, regulatory responses, and evolving perceptions regarding its impact. For the first 20 years, until around 2012, it received significant attention from authorities and maize producers across Europe.

However, it eventually became an established pest, perceived as manageable due to its monophagous nature, feeding almost exclusively on maize (*Zea mays* L.), which led to it being largely overlooked by responsible institutions. Nevertheless, sporadic studies conducted after 2012 have shown that the pest’s activity still fluctuates, with alternating periods of low and high prevalence. Some regions, particularly in Southeastern Europe [23,24,25,26,27,28,29,30,31], have reported high frequencies, while others have experienced eradication followed by re-emergence. A notable example is Belgium, where according to the National Plant Protection Organization (NPPO), the pest was present until 2012, then eradicated, only to reappear in some areas as of 2023 [32,33].

The adult stage, which is migratory and responsible for reproduction, causes damage that can affect pollination. It feeds on leaves as well as on both male (pollen-producing tassels) and female (silks) inflorescences of maize. The silks, which are essential for fertilization, can be damaged by adult feeding, potentially reducing kernel development and grain yield [34,35,36]. The species demonstrates relatively good adaptability to environmental conditions [37].

The economic impact has been interpreted differently by researchers. Some focus on the larvae, others on the adults, and still others on the combined impact of both stages [38,39]. Unlike in its native range (the United States), where adult feeding on maize silks can result in yield losses of up to 40%, estimates for Europe suggest losses of up to 10% in regions with high economic importance and advanced pest control systems [40,41]. Some studies argue that it is no longer a major pest in Europe [42]. However, in Eastern Europe, where maize is cultivated on large scales comparable to the U.S., there is concern about a strong resurgence, especially in the context of future climate change.

Regardless of whether the focus is on adults or larvae, the damage is similar. The species has only one generation per year [43], and it currently develops exclusively on maize [44]. Removing adults prevents egg-laying, while targeting larvae (which feed on roots) disrupts the development cycle. However, these control strategies are considered secondary when compared to environmental factors and temperature fluctuations, which play a more critical role in influencing female fertility, adult feeding behavior, and the overwintering success of eggs [45].

In the context of ongoing climate change, Europe has experienced a sharp increase in temperatures over the past decade. According to the European Environment Agency (EEA), the continent’s average annual temperature has risen by approximately 2.12 to 2.19 °C above pre-industrial levels, higher than the global average [46]. In 2024, Europe recorded its highest-ever temperatures, particularly in Central, Eastern, and Southeastern regions, according to the World Meteorological Organization (WMO) [47]. Clearly, rising surface temperatures can disrupt insect life cycles, most often leading to population increases. It is well established that warmer temperatures can accelerate pest development. According to Alfizar and Siti Shofiya Nasution (2024), elevated temperatures may shorten insect life cycles and increase reproductive success, thereby boosting population growth [48].

Temperature influences food consumption, distribution, population size, and migration, factors that are particularly relevant for invasive species such as *Diabrotica virgifera virgifera* in Europe [49]. These biological processes are strongly modulated by thermal conditions, and as temperatures rise, the pest’s capacity to colonize new territories is enhanced. Warmer climates can also reduce generation time, increase survival rates, and extend the period of adult activity, which together facilitate a more rapid and extensive spread across suitable habitats [49,50,51].

The existence of regional climate models (RCMs) covering long timeframes, up to 80 years, demonstrates a linear relationship between rising temperatures and the rate of thermal accumulation (degree-days), which supports faster development of the insect [50,51,52].

In reviewing models for this invasive species at a continental level, we found that available data were either global in scope, regionally limited, or outdated. Therefore, we deemed it necessary to update the prediction of its spread under current climatic conditions, particularly given that the species has regained attention in Europe since 2021 [53,54].

In this context, the present work aims to provide a mapped representation of the distribution of DvvLC in correlation with temperature deviations across Europe from 1992, the year of its first detection, to 2024. Additionally, we incorporate projections to forecast its potential expansion up to the year 2054, based on current trends and climate data.

This information could prove valuable to maize producers and plant health authorities in currently unaffected areas, encouraging increased surveillance and the implementation of regular monitoring programs.

## 2. Materials and Methods

### 2.1. Collecting Data on the Current Distribution of DvvLC

Data were collected from authoritative sources such as the EPPO Global Database [21] and the CABI Digital Library [55], accessible at https://gd.eppo.int/taxon/DIABVI/distribution (accessed on 7 July 2025) and https://www.cabidigitallibrary.org (accessed on 13 July 2025). The full scientific name, *Diabrotica virgifera virgifera* Le Conte, was used to search and verify the accuracy of the records.

**Figure 1:**
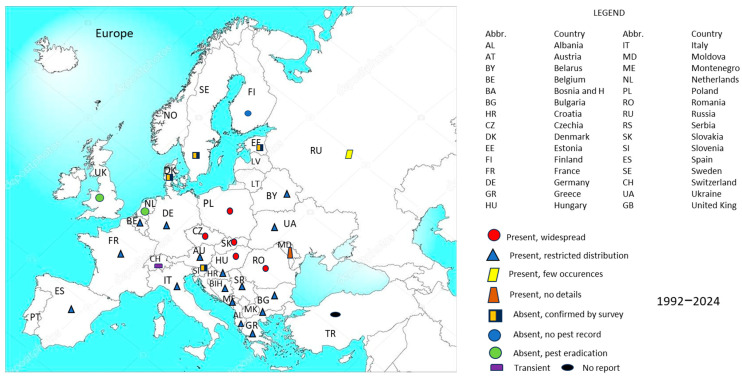
Map showing the current distribution of the species DvvLC in Europe, based on data from scientific literature and the EPPO database. The analysis period: 1992–2024, with 1992 marking the first report of the species in Europe (in Serbia).

To develop the geographical distribution map of the species (Figure 1), we considered data from 28 European countries with reported monitoring activities up to the year 2024. The reported presence status in each country was used for the analysis, covering the period from the first detection in Europe (1992) through 2024.

Each country’s status was coded using conventional symbols commonly employed in such distribution maps, indicating whether the species was: absent, present and widespread, present with restricted distribution, present with few occurrences, absent due to eradication, or considered transient. For countries with extensive territory or wide ecological zones, such as Russia and Ukraine, the presence data refer to the western regions where maize cultivation is significant and where official records of pest detection were available, primarily between 22 and 32° E longitude.

In regions where maize is scarce or not yet widely cultivated, *DvLC* adults have been reported to feed on alternative host plants, including members of the Cucurbitaceae, Fabaceae, and Heliantheae families. This facultative feeding behavior may support temporary survival and dispersal outside traditional maize zones, particularly during initial stages of invasion or in transition zones.

### 2.2. Collection of Current Corn Cultivation Data

These data come from official sources and publicly available datasets from the past two years (2022–2024), including Eurostat (eurostat.ec.europa.eu (accessed on 13 July 2025)) [56], FAOSTAT (Food and Agriculture Organization of the United Nations) (faostat.fao.org (accessed on 12 July 2025) [57], and the USDA Foreign Agricultural Service (FAS) [58], particularly for major corn-producing countries such as Romania, Ukraine, and Hungary. Additional information was gathered from national Ministries of Agriculture (e.g., MADR—Romania) [59].

For Nordic countries where detailed corn cultivation data was unavailable, estimates were made based on climate conditions, latitude, total arable land area, and Eurostat reports confirming the absence of significant commercial corn production.

**Figure 2:**
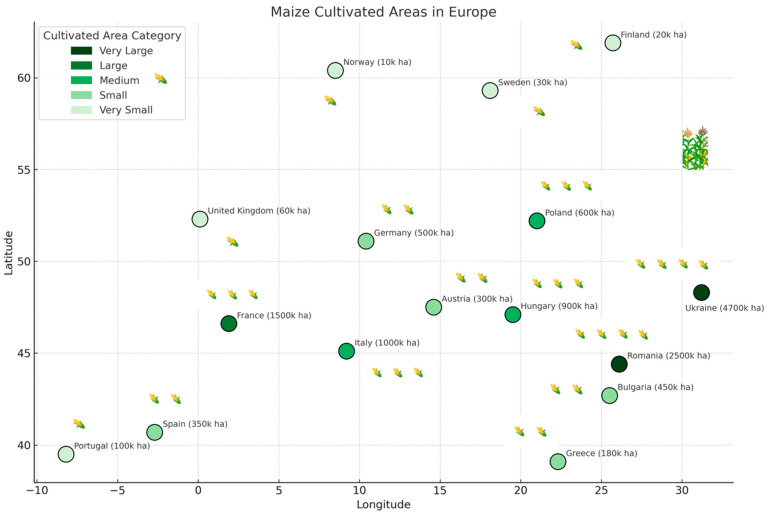
Simplified map showing the current zonal areas cultivated with maize in Europe, within defined longitude and latitude boundaries. The areas are expressed in thousands of hectares and color-coded in shades of green according to their size; Detailed legend: dark green—very large areas (>2000 k ha); medium green—large or medium areas (1000–500 k ha) and light and very light green—small or very small areas (<500 k ha).

The method for estimating the corn cultivation area involved calculating the average annual values from the last two years and converting these to area figures expressed in hectares (ha) or thousands of hectares (k ha) for use in the map. For instance, Romania, with an estimated 2.5 million hectares of corn, is represented as 2500 k ha on the map (Figure 2).

The calculation followed a simplified estimation formula (according of FAOSTAT), commonly used in such analyses:

Final area (k ha) = Average of the last 2 years from official sources (rounded to the nearest tenth).

The average geographical coordinates of the analyzed regions were grouped into categories based on the size of the cultivated maize area, as follows: intensively cultivated areas: countries with very large or large maize cultivation areas, defined as >1000 thousand hectares (e.g., 48.3° N, 31.2° E; 44.4° N, 26.1° E; 46.6° N, 1.9° E; 45.1° N, 9.2° E; 47.1° N, 19.5° E); moderately cultivated corn areas: countries with medium-sized maize areas, defined as 500–1000 thousand hectares (e.g., 51.1° N, 10.4° E; 52.2° N, 21.0° E; 40.2° N, 3.7° W; 42.7° N, 25.5° E; 47.5° N, 14.6° E); weakly cultivated corn areas: countries with small or very small maize areas, defined as <500 thousand hectares (e.g., 39.1° N, 22.3° E; 39.5° N, 8.2° W; 52.3° N, 0.1° E; 59.3° N, 18.1° E; 61.9° N, 25.7° E; 60.4° N, 8.5° E). For countries with large territorial extent (e.g., Russia and Ukraine), coordinates correspond to regions where the pest was confirmed: approximately 55.0° N, 37.0° E for western Russia and 48.0° N, 31.0° E for central Ukraine.

### 2.3. DvvLC in Correlation with Temperature Deviations (1992–2024)

In accordance with data from the Copernicus Climate Change Service (CCCS) [60] and based on the European Climate Assessment & Dataset (ECA&D) and its gridded version E-OBS (https://www.ecad.eu/download/ensembles/download.php (accessed on 14 July 2025)) [61], detailed maps of temperature anomalies (deviations) in Europe were created. These maps correspond to decadal periods and align with the reported detection and spread of the invasive pest DvvLC.

The temperature anomalies represent deviations from the average reference period (1991–2020) and highlight regions experiencing significant warming. Starting from 1992, the year of the first detection in Europe, through to 2024, periodic temperature deviation maps were produced for six-time intervals: 1991–1992; 1992–2002; 1992–2012; 1992–2020; 1992–2022 and 1992–2024.

The temperature deviations were calculated as annual anomalies from the average reference period (1991–2020), considering full calendar years. However, due to the biological specificity of DvvLC, which requires accumulated heat for development and dispersal, the correlation was also analyzed in relation to the warm season (April–September), with a focus on periods in which average temperatures exceeded 15 °C. This threshold reflects the minimum thermal requirement for adult beetle activity.

The correlation with DvvLC distribution was assessed by overlapping the annual presence/absence data with the corresponding spatial temperature deviation maps in a GIS environment. Regions showing both significant warming trends and confirmed pest presence were highlighted, indicating a spatial and temporal association between increased temperatures and the pest’s observed spread.

### 2.4. Predictability Mechanisms of DvvlC Spread

To realistically predict the spread of DvvLC in Europe under future climate conditions, we used the current distribution data as described in Section 2.1. The timeframes for spread estimation were based on typical patterns for invasive species: Medium term (5–30 years): Characterized by moderate climate change and regional expansion, aligned with IPCC intermediate climate scenarios; Long term (>30 years): Assumes more extreme climate scenarios and the emergence of new risk zones.

For this purpose, we used a simplified modeling approach, including prediction maps and risk scenarios, based on established thermal thresholds for DvvLC development, specifically a minimum of 12.5 °C for adult beetle activity in temperate zones and 9 °C for establishment in colder climates [54].

To determine potential regions suitable for DvvLC establishment and spread, the modeled temperature data were compared to these biological thresholds. Specifically, we identified areas where the projected monthly or seasonal mean temperatures exceed the required limits (≥12.5 °C or ≥9 °C) for at least two consecutive months during the growing season. This approach ensured alignment between biological requirements for post-winter activity and climatic suitability in the forecasted periods.

The models incorporated both minimum and maximum temperature limits and applied climate projections (mean annual and extreme temperatures) for the next: 10 years (up to 2034); 30 years (up to 2054). These projections followed IPCC scenario pathways SSP2-4.5 and SSP3-7.0.

### 2.5. Elements and Models Used in Developing of Risk Maps

To generate medium- and long-term risk maps, we applied a logistic growth model to estimate the expansion of infested areas over time. Using QGIS software (QGIS Development Team, version 3.22, 2021), GIS-based prediction maps were created for specific future milestones, 2034, 2054, and 2074. These maps incorporated: projected temperature anomalies; the current distribution map (2024) and an overlay method to identify comparative expansion risk. Maps were created using longitude and latitude grids, with areas marked by predicted colonization risk. For spatial reference, the central geographical coordinates (centroids) of each country were used, as defined by the World Countries Centroids dataset (WCC) [62].

To assess the risk of DvvLC expansion in countries with current or potential presence, we considered the known thermal biology of the species: a lower developmental/activity threshold and an optimal range for development (approximately 18–32 °C). In addition, completion of one generation requires the accumulation of a species-specific sum of heat units (growing degree days, GDD) above a base temperature, from post-diapause egg development to adult emergence and oviposition [63].

## 3. Results

Currently and predictive expansion of the DvvLC species in Europe is divided into 2 stages, before (as distribution maps based on existing data) and after 2024 (through risk maps).

### 3.1. Current Expansion of the DvvLC Species Since First Reporting, in Relation to Temperature Deviations

#### 3.1.1. Expansion of DvvLC Between 1992 and 2002

Following its first detection in Europe, in Serbia (then part of former Yugoslavia) in 1992 [64], *DvvLC* began expanding both westward and eastward. Within a decade, it had spread to multiple neighboring countries: Croatia (1995) [5], Hungary (1995), Romania (1996) [3,4], Bosnia and Herzegovina (1997) [65], Bulgaria and Italy (1998) [6,66], Slovakia and Switzerland (2000) [6], and Austria and France (2002) [6,67].

The case of France is particularly notable, as the detection occurred far from previously affected regions, suggesting non-continuous, human-assisted dispersal, possibly via air transport, whereas other reports align with gradual natural spread across maize-producing regions.

**Figure 3:**
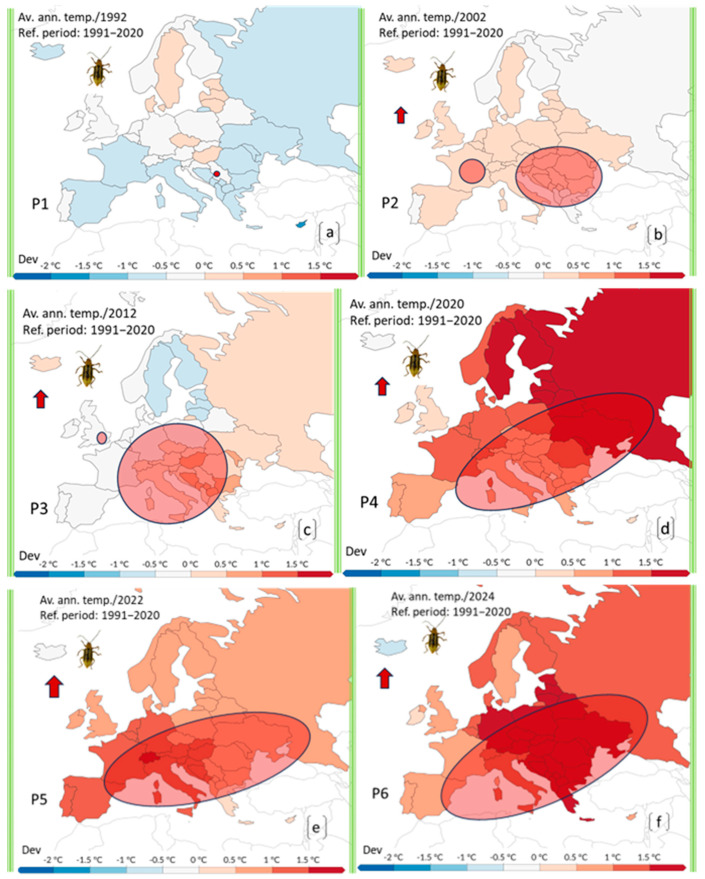
Multiple detailed maps of annual temperature anomalies across Europe were compiled to match the reporting and expansion phases of the invasive pest (DvvLC), from its first detection (1992) to 2024. The maps reflect deviations from the 1991–2020 reference period (RP) and correspond to six cumulative time intervals: (**a**) 1991–1992, (**b**) 1992–2002, (**c**) 1992–2012, (**d**) 1992–2020, (**e**) 1992–2022, (**f**) 1992–2024. Ellipses indicate the approximate geographic range of reported presence or expansion of the pest during each interval. Temperature deviation is color-coded, from blue (cooler) to deep red (strong positive anomaly). The aim is to illustrate how the pest’s expansion relates to shifting thermal conditions across Europe.

Overall, the pest expanded in all cardinal directions from the initial outbreak, with a clear trend toward Western Europe. This early phase of dispersal (1992–2002) took place in areas with minimal temperature anomalies compared to the 1991–2020 reference period, generally not exceeding +0.5 °C (Figure 3a,b).

It is important to note that these projections are based solely on climatic suitability derived from temperature thresholds relevant to DvvLC biology. The model does not account for other potential drivers of spread, such as trade, transportation routes, or passive dispersal via infested plant material. As such, the maps reflect potential climatic limits, rather than the full dispersal dynamics of the pest. This climate-envelope modeling approach has been commonly used in pest risk mapping under climate change scenarios [68].

#### 3.1.2. Expansion Twenty Years After the First Report

By extending the analysis to the period 1992–2012 (P3) (Figure 3c), it becomes clear that DvvLC had significantly expanded its range westward, covering nearly twice the area affected in the previous decade.

The pest had become widespread in countries with previously confirmed presence, such as Serbia, Croatia, Hungary, Romania, Bulgaria, Slovakia, Slovenia, Bosnia and Herzegovina, and Italy, and had expanded further in Austria [66]. Additionally, isolated detections were reported in France, Germany, Switzerland, Poland, Russia, the Czech Republic, Belgium, and the Netherlands [67], with occasional interceptions also recorded in the United Kingdom [69].

This phase of expansion was accompanied by a slight increase in temperature deviations compared to the reference period. The DAT (Deviations from Average Temperatures) rose to 0.5–1.0 °C, with a few isolated locations reaching up to 1.0 °C (Figure 3c).

#### 3.1.3. DvvLC Expansion Thirty Years After the First Report

During the P4 period (1992–2020), the pest continued to spread both southwestward and eastward, to a relatively equal extent (Figure 3d). According to the EPPO Database [21], the distribution area by 2020 had clearly expanded compared to previous periods. Countries maintaining a positive status with widespread presence included Hungary, Romania, Slovakia, Slovenia, Serbia, Bosnia and Herzegovina, and Bulgaria. Restricted distribution was observed in Belgium, Germany, Italy, Poland, Russia, and Belarus [21]. In the case of countries such as Moldova and Russia, official confirmations of initial detections were reported later, and were accordingly documented [18].

This period saw the most significant expansion of *DvvLC*, coinciding with 2020 being one of the warmest years recorded in Europe. The spread closely correlated with temperature anomalies, as DAT values rose dramatically, ranging between 1 °C and 2.5 °C (Figure 3d).

#### 3.1.4. DvvLC’s Expansion in 2022

Following the sharp global temperature rise in 2020, the expansion observed during the P5 period (1992–2022) (Figure 3e) was slower. This phase also reflected a partial stagnation or slight regression in the overall invaded area compared to the previous period. The pest advanced southwestward, where DAT values reached or exceeded 1.5 °C, while its spread towards Eastern Europe appeared to stall, with temperature deviations falling back to around 0.5 °C. This climatic trend may explain the first confirmed presence of *DvvLC* adults in Spain in 2021 [19].

#### 3.1.5. DvvLC’s Expansion in 2024

In the P6 period (1992–2024), marking 34 years since the first detection, a more pronounced expansion was observed in all directions, particularly towards the south and west, along with a slight shift to the north (Figure 3f). The total area occupied by the pest increased, and presence statuses were updated as follows: Widespread distribution in Hungary, Romania, Poland, the Czech Republic, and Slovakia. Restricted distribution in Serbia, Albania, Austria, Belgium, Belarus, Bulgaria, Croatia, France, Bosnia and Herzegovina, Germany, Italy, Greece, Montenegro, Switzerland, and Spain. Isolated occurrences reported in Slovenia and Russia [21]. Due to insufficient data, Moldova was excluded from this analysis.

In 2024, notable temperature shifts back toward the east and north were recorded, creating potential confusion in the migration and expansion patterns of DvvLC. With DAT values increasing in the initial outbreak zone (1.5–2.5 °C) and decreasing in Western Europe (down to 0.5 °C) (Figure 3f), the future trajectory of spread may become less predictable and more chaotic.

### 3.2. Maps Predicting the Expansion of the DvvLC Species in Correlation with the Extension of Corn Areas

Two sets of predictive maps were generated based on projected climate change, specifically considering gradual increases in average temperature by 0.5 °C every 5 years, potentially reaching 2.6 °C by 2054 (Table 1).

**Figure 4:**
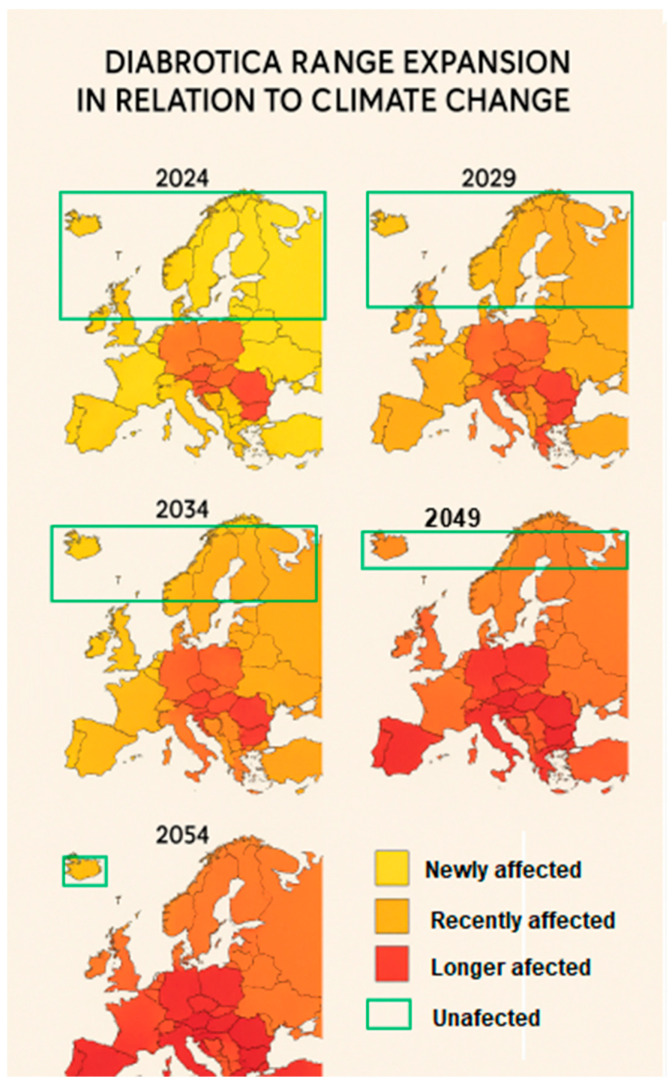
Projected expansion of DvvLC in Europe areas under climate change scenarios for the years 2024, 2029, 2034, 2049, and 2054. Categories: Newly affected (yellow), Recently affected (orange), Longer affected (red), and Unaffected (green).

In Figure 4, the first set of five predictive maps (developed using a logistic growth model) illustrate the forecasted spread of *DvvLC* across: Short-term: 2024–2029, Medium-term: 2024–2034 and 2024–2039 and Long-term: 2024–2054. These projections are based on temperature trends, both annual averages and extremes, provided by the Copernicus Climate Change Service (CCCS), and account for *DvvLC*’s biological thresholds and favorable development conditions.

In parallel, maize cultivation is highly influenced by temperature conditions. Maize typically requires a minimum soil temperature of 10 °C for germination and optimal growth temperatures ranging between 24 °C and 30 °C during the growing season. Prolonged exposure to temperatures above 35 °C, especially during pollination, can significantly reduce yields due to heat stress and pollen sterility. Additionally, a growing season with accumulated heat units (growing degree days—GDD) between 1000 and 1400 GDD (base temperature 10 °C) is generally necessary for full crop maturity, depending on hybrid selection. These parameters were considered when analyzing the projected northward shift in suitable maize cultivation areas under climate change scenarios.

Based on the analysis of the generated prediction maps (Figure 4), our results indicate that in the short term (by 2029), DvvLC may reach the southern regions of Northern Europe, where average air temperatures are projected to rise by approximately +1.6 °C (Table 1). Such thermal conditions would not only favor maize cultivation but also enhance the development and overwintering survival of the pest. In the medium term, with projected temperature increases ranging from +1.8 °C to +2.4 °C (Table 1), the likelihood of successful maize cultivation and DvvLC establishment may extend into the northern half of the Scandinavian Peninsula. Nevertheless, it is essential to underline that thermal suitability does not automatically translate into agronomic feasibility. In these regions, maize expansion remains conditional on other critical factors, including soil composition, water availability, and socioeconomic or policy-related constraints. In the long term, with average temperatures expected to exceed +2.4 °C (Table 1), DvvLC may adapt to almost all of Northern Europe under favorable climatic conditions, while maize cultivation expansion would still depend on broader environmental and economic feasibility.

It is important to note that these long-term projections are based on predictive climate models and should therefore be regarded as scenarios rather than confirmed outcomes.

The multi-map series (Figure 4) illustrates the progressive northward shift in DvvLC-infested areas over the period 2024–2054, with newly affected, recently affected, and longer affected regions being highlighted. In parallel, maize cultivation zones are projected to shift northward as well, although with more uncertainty due to agronomic and socioeconomic limitations.

In the short-term outlook (2024–2034), Central and Eastern Europe are expected to remain the core maize production regions, with Romania, Hungary, and Poland continuing as leading producers. Northern European countries, such as Germany and the Netherlands, may begin to incorporate maize more frequently into crop rotations, likely due to lengthened growing seasons caused by rising temperatures. By 2044, maize cultivation is projected to expand into Denmark and southern Sweden, while southern Europe (e.g., Spain and Italy) may experience reduced yields as a result of heat stress and drought.

By 2054, maize cultivation could become established in parts of southern Scandinavia under newly favorable climatic conditions, whereas southern Europe will likely become less suitable for maize due to extreme weather and increasing water stress. Central Europe is expected to maintain relatively stable cultivation zones, with only moderate changes.

Overall, while temperature trends suggest that maize cultivation could extend northwards in the coming decades, these projections should be interpreted with caution. Additional constraints, such as soil properties, water availability, and especially the economic competitiveness of maize relative to other crop, may limit its actual expansion. Similarly, pest establishment depends not only on thermal thresholds but also on critical biological phases such as egg overwintering, larval survival, and synchronization with host plant phenology.

### 3.3. Indirect Correlations Between Temperature Increase, DvvLC Spread, and Expansion of Maize Cultivation Areas over a 30-Year Predictive Period

**Figure 5:**
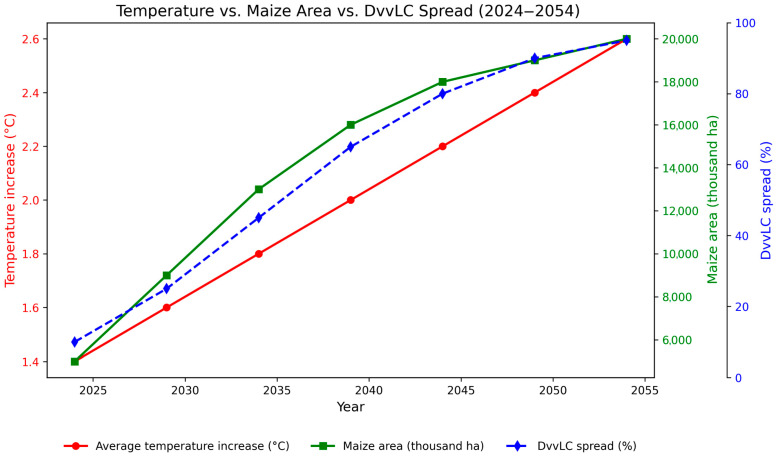
Temperature increase (°C), maize cultivated area (thousand hectares), and DvvLC spread (%) in Europe during the period 2024–2054. The left Y-axis (red) shows the average temperature increase (°C). The right Y-axis presents both the maize cultivated area (green, thousand ha) and the DvvLC spread (blue, %).

Figure 5 illustrates the indirect correlation between increasing temperatures, the spread of *Diabrotica virgifera virgifera*, and the expansion of maize cultivation areas across Europe during the 30-year predictive period (2024–2054). The model indicates a gradual increase in average temperature from 1.4 °C to 2.6 °C, which is accompanied by a marked northward expansion of DvvLC, with the pest potentially infesting up to 95% of suitable regions by 2054.

In parallel, the maize cultivation area is projected to increase substantially, from about 6 million hectares in 2024 to approximately 20 million hectares by 2054, reflecting both the crop’s adaptation to warmer climates and the anticipated expansion into northern and central European regions. This expansion overlaps with the predicted spread of DvvLC, highlighting the strong link between climate-driven crop expansion and pest proliferation.

The graphical correlation emphasizes that while rising temperatures create new opportunities for maize production, they simultaneously accelerate the spread of DvvLC into previously pest-free areas, thereby increasing the risk of yield losses and management challenges under future climate scenarios. The species predicted range expansion also considers the thermal thresholds required for adult beetle activity and dispersal, including a minimum of 12.5 °C in temperate zones and 9 °C in colder climates [70,71]. These thresholds were used to estimate areas where environmental conditions would allow the pest to establish and persist, particularly under climate change scenarios that influence both crop viability and pest proliferation.

### 3.4. Estimating the Risk of DvvLC Expansion Based on Latitude and Longitude

Table 2 provides an estimation of the risk level for DvvLC expansion in each targeted European country, based on centroids dataset geographical location and latitude and longitude coordinates (WCC-World Countries Centroids) [62]. This approach allows for a spatially informed assessment of the likelihood of pest establishment, integrating climate suitability, maize availability, and potential dispersal routes.

Based on the analysis of geographic centroids for 29 European countries (Table 2), risk levels of DvvLC expansion were estimated for three-time horizons: 2034, 2054, and 2074. The spatially explicit assessment, grounded on latitude and longitude data from the WCC (World Countries Centroids), reveals temporal and regional variation in the predicted risk levels.

A consistent trend was observed across most of Central and Southeastern Europe, where several countries, including Bulgaria, Serbia, Greece, Italy, Romania, and Bosnia and Herzegovina, exhibited a High Risk (HR) status in all three-time frames. These regions are situated in lower latitudes and have climate conditions already favorable to DvvLC development and maize cultivation, which likely contributes to the persistent high-risk categorization.

It is noteworthy that countries with already established DvvLC populations, such as Serbia, Bulgaria, Romania, and Italy, also fall into the High- Risk category across all time horizons. This alignment supports the model’s predictive capacity and confirms that it reliably reflects present infestation patterns as well.

A second group of countries, such as Austria, France, Croatia, Slovenia, and Switzerland, displayed an increase in risk level over time. For instance, Austria transitioned from Moderate Risk (MR) in 2034 and 2054 to High Risk (HR) by 2074. Similar patterns were recorded for France and Croatia, indicating a temporal progression toward more suitable conditions for pest establishment.

In Western and Northern Europe, particularly Germany, Belgium, the Netherlands, and Poland, the risk level increased from Low (LR) or Moderate (MR) to Moderate or High by 2074. Notably, Germany and Poland progressed from Low Risk to Moderate Risk, suggesting a northward shift in potential pest distribution linked to projected climate change scenarios.

Conversely, countries at higher latitudes, such as Estonia, Latvia, and Lithuania, maintained a Low Risk (LR) status throughout the analyzed period. This stability reflects the continued climatic unsuitability of these regions for DvvLC establishment, likely due to lower thermal accumulation and shorter growing seasons.

These temporal shifts in risk levels, aligned with geographic gradients, underscore the relevance of latitude and climatic suitability in modeling potential future expansion zones of DvvLC across Europe.

**Figure 6:**
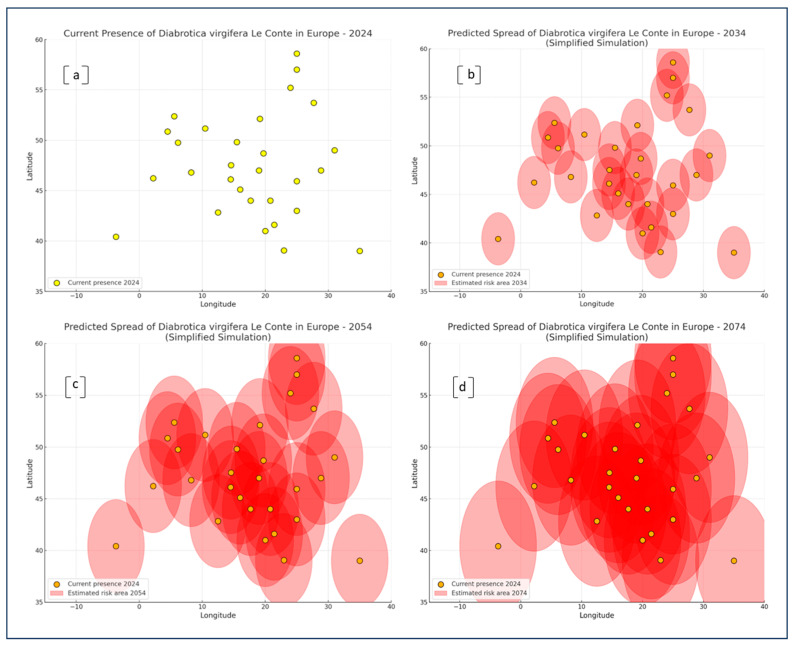
Risk area simulations were conducted using a simplified modeling approach, based on known thermal thresholds for the development of DvvLC, including both minimum and maximum temperature limits. The model integrates climate projections for annual mean and extreme temperatures across Europe, with simulations targeting three future time horizons: the next 10, 30, and 50 years (until 2074; reference year 2024). In detail: (**a**) Current presence of DvvLC in Europe in 2024, represented by yellow points indicating confirmed pest occurrences; (**b**) Predicted spread of DvvLC by 2034, showing an initial expansion of risk areas (red ellipses) based on climatic suitability, with current presence points included for reference; (**c**) Predicted spread of DvvLC by 2054, illustrating further enlargement of risk zones and increasing pest establishment potential across Central and Eastern Europe; (**d**) Predicted spread of DvvLC by 2074, projecting extensive expansion across most of Europe, with overlapping high-risk zones and widespread climatic suitability.

Figure 6 provides a spatial simulation of the predicted distribution of DvvLC across Europe over three future time frames, 2034, 2054, and 2074, relative to its current known distribution in 2024. The visual representation is based on circular buffer zones around national centroids, approximating potential risk areas of establishment based on geographical position and estimated suitability.

In Figure 6a, the current distribution (2024) is shown as discrete points, representing countries with either confirmed pest presence or conditions conducive to establishment. The distribution is primarily concentrated in Central and Southeastern Europe.

By 2034 (Figure 6b), the emergence of risk zones begins to extend northward and westward. Several new areas, particularly in Western and Central Europe, show signs of increased spatial vulnerability, though their extent remains moderate. The dispersion pattern suggests early-stage colonization potential beyond current zones.

The projection for 2054 (Figure 6c) reveals a marked expansion of estimated risk zones, forming overlapping areas that encompass much of the temperate zone of Europe. This phase shows the emergence of spatial connectivity between previously isolated risk nodes, especially along a southwest–northeast corridor running from the Iberian Peninsula through Central Europe and into parts of Western Russia.

By 2074 (Figure 6d), the estimated risk regions coalesce into a nearly continuous belt across continental Europe. This visualization underscores not only the increased geographic range but also the intensification of potential establishment areas, even in higher latitudes. Northern and Western European regions, previously at low risk, now appear substantially encompassed by projected risk zones.

Overall, these visual simulations support the hypothesis of a progressive, climate-driven spatial expansion of DvvLC, confirming that regions currently unaffected may face significantly increased establishment pressure in the coming decades.

## 4. Discussion

The spatio-temporal modeling of DvvLC distribution across Europe, as presented in this study, offers a comprehensive outlook on pest expansion potential through 2074. Integrating geospatial centroids, climate suitability, and host crop availability, the results align with the working hypothesis that both latitudinal gradients and temperature increase play a pivotal role in facilitating the pest’s potential for establishment.

Data from Table 2 provide strong support for a structured risk pattern correlated with geographic position. Countries already situated in low-latitude zones (e.g., Romania, Serbia, Bulgaria, Greece, Italy) were consistently classified as high-risk across all temporal intervals (2034–2074). These findings reinforce previous work by Ciosi et al. [43], who detailed the rapid invasion and adaptation of DvvLC within Southern Europe during the 1990s and early 2000s, and by Igrc Barčić et al. [5], who emphasized the role of favorable temperature regimes in supporting multiple generations per year. Our projections extend this paradigm longitudinally, identifying Balkan, Mediterranean, and Black Sea regions as persistent hotspots under both current [21], and future conditions.

More notably, however, the results highlight a progressive risk increase in Central and Western Europe. Countries such as Austria, Germany, Poland, Switzerland, and France—previously considered peripheral or marginal for the pest’s development, transition from Low or Moderate Risk to High Risk toward 2074. These outcomes are consistent with Schneider et al. [72], who used RCP-based climate models to predict increased habitat suitability for invasive pests across temperate Europe. Similarly, Kiss et al. [12] discussed the relationship between mean annual temperature and pest developmental thresholds, concluding that as climatic barriers weaken, colonization of higher latitudes becomes plausible.

Visual projections from Figure 6 reinforce this trend. The spatial progression from 2024 to 2074 reflects a clear northward and westward shift in climatically favorable zones. The aggregation of risk areas into broader contiguous corridors, especially across Central and Eastern Europe, suggests that previously fragmented niches may evolve into more stable pest habitats. These findings parallel the expansion scenarios simulated by Bažok et al. [42], who illustrated growing infestation pressure in countries like Hungary, Croatia, and Austria under future climate projections. Notably, our visual maps highlight areas such as the southern half of Germany, eastern France, and western Ukraine as emerging transition zones, deserving targeted monitoring.

The methodological innovation of this study, the use of centroid-based spatial buffers, provides a generalized yet scalable mechanism for cross-country comparison. While not as precise as grid-based or mechanistic species distribution models, this approach complements those used by Sivčev et al. [64], particularly for regional-scale forecasting. The relative simplicity of our model allows it to be adapted to national risk frameworks or biosecurity plans, especially in countries where pest surveillance is still in early stages.

Our results also intersect with emerging genomic and physiological findings. Coates et al. [73] recently demonstrated that DvvLC populations exhibit notable genomic flexibility, including genes associated with host recognition and environmental stress adaptation. These traits likely contribute to the pest’s resilience and capacity to establish in novel regions, a mechanism supported by our data showing increasing risk in areas previously deemed climatically marginal. In support of this, Botsch et al. [74] provided experimental evidence that DvvLC can tolerate brief periods of thermal stress exceeding 38 °C without mortality effects, expanding the understood thermal limits for population survival.

From a practical perspective, field data from recent Romanian trials [36] show increased larval survival and adult activity during 2023–2024 in locations aligning closely with the HR zones predicted by our model. These observations validate the theoretical risk scores assigned and support the model’s application to decision-making in plant protection. Moreover, Amarghioalei et al. [75] reported that previously effective chemical control methods have shown reduced efficacy, possibly linked to climate-driven phenological shifts and population dynamics.

Despite robust alignment with published literature, several findings diverge from earlier expectations. For example, Baltic countries (Latvia, Estonia, Lithuania) maintain Low Risk (LR) status in our model even through 2074. This contradicts certain niche-based models that forecast moderate suitability in these regions by mid-century [18]. However, it is plausible that microclimatic heterogeneity, shorter maize cultivation windows, and lower soil temperatures continue to function as limiting factors in these high-latitude environments.

Although temperature trends suggest that maize cultivation could become possible even in more northern regions, such as southern Sweden, it is important to acknowledge that thermal suitability alone does not guarantee agronomic feasibility. Other factor, such as soil composition, water availability, and especially the economic viability compared to traditional or more productive crops, may limit the actual expansion of maize in these areas. Therefore, projected expansion maps should be interpreted with caution and viewed as a potential thermal envelope, rather than confirmed cultivation scenarios.

In addition to summer thermal thresholds for adult activity, overwintering success of eggs and early larval development are highly sensitive to winter and early spring temperatures. Projected climate change may influence these critical phases, potentially improving survival in colder areas. Moreover, shifts in the start of the vegetation period and crop phenology must be considered, as maize growth and pest development may become desynchronized, particularly under extreme drought or soil moisture deficits.

Under future climate conditions, it remains uncertain whether maize can reach maturity in all newly suitable thermal zones. Increasing temperature extremes, combined with reduced soil moisture, may negatively impact grain development and yield, particularly in southern and continental regions. This highlights the need for integrated modeling that considers not only thermal expansion, but also water stress and crop physiology.

In terms of implications, this study adds to the growing body of work, such as the review by EPPO [21], calling for harmonized European surveillance protocols. Particularly relevant is the need to update pest monitoring thresholds and quarantine policies, considering the spatial advance into non-endemic regions. Our results also intersect with agricultural policy concerns under the European Green Deal, as they suggest that sustainable maize production may increasingly rely on adaptive IPM strategies in both core and fringe risk zones.

A limitation of the present approach is that the heat-sum requirement was represented through a persistence proxy rather than explicit degree-day calculations, due to the lack of harmonized GDD parameters across Europe. Future versions will incorporate region-specific GDD thresholds as these become available.

Future research should focus on refining pest distribution models through integration of high-resolution agroecological data, including local soil thermal properties, irrigation regimes, and crop phenology. Such improvements would allow for more nuanced predictions of establishment risk, particularly in transition areas identified in this study (e.g., Austria, southern Germany, and western Ukraine), where model outputs suggest increasing vulnerability despite current low-to-moderate pest pressure. In addition, validating centroid-based risk projections through systematic field surveys would help ground-truth spatial forecasts and reveal fine-scale ecological constraints or accelerators of spread. The incorporation of agent-based simulations that account for pest dispersal via transportation corridors, prevailing wind patterns, and cross-border trade routes could offer further insights into long-distance colonization events. Finally, expanding molecular surveillance of DvvLC populations, especially in emerging risk zones, may reveal locally evolving adaptations to temperature stress, insecticide exposure, or host plant phenology, as suggested by recent genomic studies [74]. Collectively, these directions would enhance predictive capacity and inform more precise, climate-resilient pest management strategies at both national and continental scales.

## 5. Conclusions

This study demonstrates that the spatial risk of DvvLC establishment in Europe is expected to increase substantially over the coming decades, primarily due to rising climatic suitability and continued host crop availability. Using centroid-based modeling, combined with risk classification and visual projections, the results reveal a persistent high-risk profile in Southern and Southeastern Europe, with a progressive expansion toward Central, Western, and parts of Northern Europe by mid-to-late 21st century.

The spatial simulations highlight a clear trend of northward and westward shift in potential establishment zones, consistent with broader patterns of climate-driven range expansion. These findings are further supported by recent genomic and physiological research indicating the species’ adaptability to changing thermal and environmental conditions. The overall progression of risk underlines the need to reassess existing surveillance and management frameworks, especially in transitional agroclimatic zones not previously considered vulnerable. The study offers a flexible and regionally applicable risk assessment approach that can support anticipatory pest control strategies and inform agricultural policy decisions at the continental level.

## Figures and Tables

**Table 1:** Estimated increase in average air temperatures in Europe (2029–2054), based on CCCS projections.

Year	Estimated Increase (°C) ^1^	Year	Estimated Increase (°C)
2024/+1.4 °C ^2^			
2029	+1.6 °C	2044	+2.2 °C
2034	+1.8 °C	2049	+2.4 °C
2039	+2.0 °C	2054	+2.6 °C

^1^ pre-industrial period (around 1900), as a reference period; ^2^ starting values.

**Table 2:** Estimation of the risk level for DvvLC expansion in each targeted European country, based on approximate location and geographic coordinates, over 10, 30, and 50-year time horizons.

Country	Longitude (°)	Latitude (°)	Estimated Risk		
			2034	2054	2074
Albania	20.00 (E)	41.00 (N)	HR ***	HR ***	HR ***
Austria	14.55 (E)	47.52 (N)	MR **	MR **	HR ***
Belarus	27.70 (E)	53.70 (N)	LR *	LR *	MR **
Belgium	4.47 (E)	50.85 (N)	LR *	MR **	MR **
Bosnia and Herz.	17.67 (E)	44.00 (N)	HR ***	HR ***	HR ***
Bulgaria	25.00 (E)	43.00 (N)	HR ***	HR ***	HR ***
Croatia	16.00 (E)	45.10 (N)	MR **	HR ***	HR ***
Czechia	15.47 (E)	49.82 (N)	MR **	MR **	MR **
France	2.21 (E)	46.22 (N)	MR **	HR ***	HR ***
Germany	10.45 (E)	51.16 (N)	LR *	MR **	MR **
Greece	22.95 (E)	39.07 (N)	HR ***	HR ***	HR ***
Hungary	19.00 (E)	47.00 (N)	MR **	MR **	HR ***
Italy	12.50 (E)	42.83 (N)	HR ***	HR ***	HR ***
Luxembourg	6.13 (E)	49.75 (N)	MR **	MR **	MR **
Moldova	28.85 (E)	47.00 (N)	MR **	MR **	HR ***
Netherlands	5.55 (E)	52.37 (N)	LR *	LR *	MR **
North Macedonia	21.43 (E)	41.61 (N)	HR ***	HR *	HR ***
Poland	19.15 (E)	52.12 (N)	LR ***	LR *	MR **
Romania	25.00 (E)	45.94 (N)	MR **	HR ***	HR ***
Serbia	20.80 (E)	44.00 (N)	HR ***	HR ***	HR ***
Slovakia	19.70 (E)	48.70 (N)	MR **	MR **	HR ***
Slovenia	14.50 (E)	46.12 (N)	MR **	HR***	HR ***
Spain	−4.00 (E)	40.42 (N)	HR *	HR ***	HR ***
Switzerland	8.23 (E)	46.80 (N)	MR **	HR ***	HR ***
Turkey	35.00 (E)	39.0 (N)	HR ***	HR ***	HR ***
Ukraine	31.00 (E)	49.00 (N)	MR **	MR **	MR **
Lithuania	24.00 (E)	55.20 (N)	LR *	LR *	LR *
Latvia	25.00 (E)	57.00 (N)	LR *	LR *	LR *
Estonia	25.00 (E)	58.60 (N)	LR *	LR *	LR *

* LR-Low Risk; ** MR-Medium Risk; *** HR-High Risk; (N)-Northern latitude; (E)-Eastern longitude.

## Data Availability

The authors confirm that all data in this paper are available on request from the corresponding author.
